# Low intensity trans-spinal focused ultrasound reduces mechanical sensitivity and suppresses spinal microglia activation in rats with chronic constriction injury

**DOI:** 10.1186/s42234-025-00170-z

**Published:** 2025-03-31

**Authors:** Weiguo Song, Alice Giannotti, Alexandra Bekiaridou, Ona Bloom, Stavros Zanos

**Affiliations:** 1 Feinstein Institutes for Medical Research, 350 Community Dr, Manhasset, NY 11030 USA; 2 The Biorobotics Institute and Department of Excellence in Robotics and AI, Scuola Superiore Sant’Anna, Pisa, Italy; 3 Elmezzi Graduate School of Molecular Medicine, 350 Community Dr, Manhasset, NY 11030 USA

**Keywords:** Trans-spinal focused ultrasound stimulation, Noninvasive neuromodulation, Von Frey threshold, Pain, Flow cytometry, Microglia

## Abstract

Low intensity, trans-spinal focused ultrasound (tsFUS) is a noninvasive neuromodulation approach that has been shown to modulate spinal circuit excitability in healthy rats. Here, we evaluated the potential of tsFUS for alleviating neuropathic pain by testing it in a chronic constriction injury (CCI) model. Male rats underwent CCI of the left sciatic nerve and then received tsFUS (2 kHz pulse repetition frequency; 40% duty cycle) or sham stimulation, targeted at spinal segment level L5 for 3 min daily over three days. As expected, CCI causes significant reduction of von Frey Threshold (vFT), a measure of mechanical sensitivity. We found that tsFUS treatment is associated with increased vFT compared to sham; this increase persists beyond the duration of treatment, through days 4 to 23 post-CCI. In spinal cords of tsFUS-treated animals, counts of spinal microglia (Iba1 + cells) and of activated, pro-inflammatory microglia (Iba1 + /CD86 + cells), are reduced compared to sham-treated animals. This reduction in microglia counts is limited to the insonified side of the spinal cord, ipsilateral to CCI. These findings suggest that tsFUS may be a promising approach for treatment of neuropathic pain at early stages, possibly by attenuating the development of microglial-driven inflammation.

**Supplementary Information:**

The online version contains supplementary material available at 10.1186/s42234-025-00170-z.

## Background

Chronic neuropathic pain (NP) impacts up to 10% of the general global population and pharmacological therapies are frequently fail to effectively manage pain symptoms and can lead to various side effects, some of which may be severe or addictive (Vadivelu et al. 2015), which motivates a search for non-pharmacological treatment modalities (di Biase et al 2021). NP is believed to be initiated by injury to the somatosensory peripheral nervous system and involves a persistent inflammatory response (Costigan et al. 2009). It is unclear how NP was developed after injury. Pro-inflammatory cytokines play an important role in regulating NP following peripheral nerve injury or spinal cord injury (Detloff et al. 2008; Fregnan et al. 2012). After injury, microglial cells release pro-inflammatory molecules (Li et al. 2021) and neuroinflammation has been linked to pain behaviors (Nishihara et al. 2020; Wendimu and Hooks 2022). Suppressing pro-inflammatory cytokines can alleviate hyperalgesia in rats with chronic constrictive injury (CCI), a common preclinical model of NP (Liu et al. 2017). Thus, modulating the inflammatory response of spinal microglia may be a strategy for NP treatment (Sun et al. 2022).

Various neuromodulation methods have been tested in the treatment of NP, with some success and with some notable drawbacks. Noninvasive approaches for NP mainly use electrical or transcranial magnetic stimulation of the brain or spinal cord, with minimal research on spinal cord focused ultrasound stimulation (FUS) (Joosten and Franken 2020; Xiong et al. 2022). Transcranial magnetic and direct current stimulation of the primary motor cortex, despite their generally low spatial resolution, have shown promise in the treatment of NP, even though evidence for effectiveness has sometimes been conflicting (Young et al. 2014; Xiong et al. 2022). For example, a 10-year follow-up study of patients treated with electrical stimulation of the spinal cord and/or peripheral nerves found that significant long-term pain relief was not always achieved, highlighting the need for further clinical trials (Long et al. 1981; Yang et al. 2022). In addition, in most cases, spinal cord stimulation is delivered invasively, posing risk of surgical complications (Hasoon et al. 2024). Recently, high-intensity FUS has been tested as a therapeutic option for chronic neuropathic pain through ablation of thalamic pain centers (di Biase et al. 2021; Ahmed et al. 2024; Ishaque et al. 2024) or by blocking peripheral nerve conduction (Lee et al. 2015). However, it poses risks for non-reversible damage, with possible neurological consequences that are yet undetermined. Low-intensity FUS, a noninvasive method for neuromodulation with improved spatiotemporal resolution and penetration depth, has recently gained interest for targeted peripheral neuromodulation at the level of specific organs (Downs et al. 2018; Kubanek 2018; Cotero et al. 2019; Cotero et al. 2022; Zachs et al. 2019; Liss et al. 2021; Ahmed et al. 2022; Zanos et al. 2023; Zafeiropoulos et al. 2024), or central brain areas (Kubanek 2018; Murphy et al. 2024). In the treatment of NP, low intensity FUS targeting dorsal root ganglia was shown to reduce pain symptoms in preclinical models (Liss et al. 2021). Neuromodulation effects of FUS have also been tested directly on isolated nerves (Guo et al. 2022) or during behavior (Murphy et al. 2024). Surprisingly few studies have tested low intensity FUS modulation of the spinal cord for the treatment of NP. Recently, we (Song et al. 2024) and others (Liao et al. 2021; Kim et al. 2022) used trans-spinal FUS (tsFUS) to modulate neuronal excitability and synaptic transmission in the spinal cord. In healthy rats we found that tsFUS suppressed the proprioceptive, monosynaptic H-reflex and reduced nociceptive, polysynaptic windup activity associated with slow C-afferent fibers (Song et al. 2024).

Because tsFUS could modulate excitability of the spinal circuit or impact non-neuronal intraspinal cells, including microglia (Grewal et al. 2023; Li et al. 2024), it may have a therapeutic role in NP. Here, we investigated whether sensitivity to mechanical stimuli is altered when tsFUS is delivered early after CCI, a common model of chronic NP. We also examined possible effects of tsFUS on intraspinal microglial cells of animals with CCI. We found that early tsFUS treatment in rats reduced mechanical sensitivity and activation of spinal microglia induced by CCI, supporting additional studies of tsFUS as a therapeutic option to treat NP.

## Methods

### Animal preparation

The effects of tsFUS on the NP induced by CCI were examined in 40 male Sprague–Dawley rats (250–320 g). All experiments were approved by the Institutional Animal Care and Use Committee of The Feinstein Institutes for Medical Research.

### Chronic constriction injury (CCI) model

CCI is an established rat model of chronic NP and has been proven useful for approximating aspects of human NP (Bennett and Xie 1988). CCI was performed as in (Bennett and Xie 1988). Under isoflurane (3%) anesthesia, the left sciatic nerve was exposed by making a skin incision between the gluteus and biceps femoris muscles. Three chromic gut ligatures were tied loosely around the sciatic nerve at 1 mm intervals proximal to the trifurcation of the sciatic nerve, to just occlude but not arrest epineural blood flow. The wound was closed with sutures in the muscle and staples in the skin. Animals were then allowed to recover from surgery for 3 days.

### Von Frey test

Mechanical hypersensitivity indicated by paw withdrawal thresholds to von Frey filament stimuli have been used to study mechanisms of pain in animals and shown to be consistent with the clinical experience of pain (Gregory et al. 2013). A significant von Frey threshold difference indicates a measurable change in mechanical sensitivity, which correlates with changes in subjective pain scores in patients with NP (Keizer et al. 2007). To assess the mechanical hypersensitivity blindly, all the cage cards were covered before performing the von Frey test, and rats were placed on an elevated mesh platform within a transparent cylinder. Prior to behavioral testing, rats were habituated to the experimental apparatus for 30 min. The mechanical withdrawal threshold was then measured using a dynamic aesthesiometer (Ugo Basile, Italy). The aesthesiometer applied controlled mechanical pressure directly beneath the mid-plantar surface of the hindpaw. The force applied has a cut-off limit of 150 g. Two measurements were taken with a 5-min interval between them. The average of these measurements was calculated to determine the mechanical withdrawal threshold. This threshold indicates the level of sensitivity or response to mechanical stimulation in the hindpaw.

### Trans-spinal focused ultrasound stimulation (tsFUS)

**Fig. 1:**
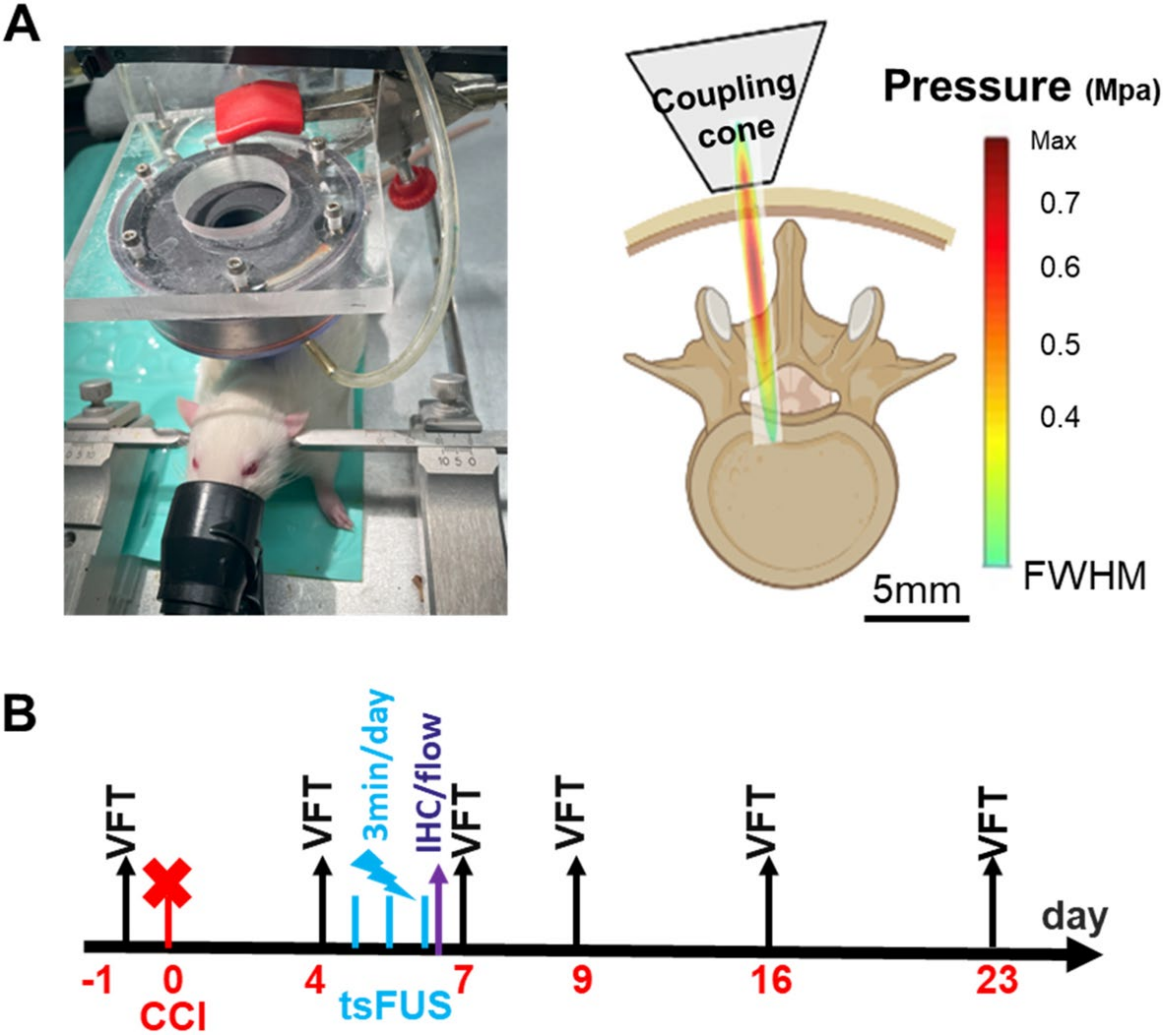
Experimental setup and timeline. **A** Trans-spinal FUS (tsFUS) was transcutaneously targeted at the L5 segment level of the spinal cord. The color map shows the ultrasound field from the transducer within the spinal cord. **B** In all animals, treatment with tsFUS or sham stimulation was administered once daily for 3 min at days 4, 5 and 6 after chronic constriction injury (CCI). In a behavioral cohort (*N* = 6 tsFUS, *N* = 6 sham), von Frey threshold (VFT) testing was conducted 1 day before CCI (baseline), and at 4, 7, 9, 16 and 23 days after CCI. Some animals were sacrificed for immunohistochemistry (IHC: *N* = 5 for sham CCI, CCI + tsFUS sham, and CCI + tsFUS) or flow cytometry (FC: *N* = 5 for sham CCI, *N* = 4 for CCI + tsFUS sham and CCI + tsFUS), on day 6 after CCI. The cone of the sketch represents only the exit tip of the physical coupling cone

tsFUS was applied as we have done previously (Song et al 2024). Briefly, anesthesia was initiated in an induction chamber with 4–5% isoflurane and maintained with 1–1.5% isoflurane. Rats were then placed on a table in a prone position, with normal body temperature maintained using a water heating pad. After shaving and further hair removal with Nair, ultrasound conductive adhesive gel was applied between the skin and the tsFUS probe. The tsFUS probe was targeted on one side of the spinal cord, slightly off the midline (half maximum amplitude of 1.8 mm laterally and 12 mm in the depth direction), and applied transcutaneously. The treated region (L5 spinal segment), which is between T13 and L1 of the rat spinal vertebra, was marked and stimulated for 3 min daily for 3 days. Our recent study revealed that this region exhibits the most optimal response to sciatic nerve stimulation (Song et al. 2024). The FUS pulse repetition frequency (PRF) was set to 2 kHz, and the duty cycle was set to 40%. tsFUS treatment was delivered for three minutes daily for three consecutive days Fig. 1.

### Immunohistochemistry

To study the potential effects of tsFUS on microglia, immunohistochemistry (IHC) was performed on the spinal cord. Three days after the end of tsFUS treatment (six days after CCI), rats were perfused with phosphate-buffered saline (PBS) and 4% paraformaldehyde, the L4-L6 spinal segments, where tsFUS irradiated, were isolated and fixed in 4% PFA overnight. Sequential cross-Sects. (10 µm thickness) of the spinal cord were cut by cryostat. Sections were incubated overnight at + 4 °C with the primary goat anti-mouse antibody anti-IBA1 (1:500, Abcam, ab5076) diluted in blocking solution of TBST. The following day, the sections were rinsed with TBST and incubated with donkey anti-goat 647 secondary antibody (1:500, Abcam, ab150131) for two hours at room temperature. Micrographs were obtained using a fluorescence microscope (BZ-X800, Keyence, USA). The number of cells was determined from both sides of the dorsolateral area of the spinal cord (from the central canal) on each slide using ImageJ software (https://imagej.nih.gov/ij/) and the average was calculated from three slides per animal.

### Flow cytometry

After dissection, the L4 to L6 lumbar spinal segments, where tsFUS irradiated, were carefully dissected and separated down the midline. Ipsilateral and contralateral spinal cord segments were processed independently and placed in 3 ml of ice-cold Hank’s Balanced Salt Solution (HBSS), where they were mechanically disrupted by rapid chopping with a scalpel blade. Each sample was digested with 3 ml of Accutase (Thermo Scientific, A1110501) and incubated at 37 °C for 10 min. After incubation, enzymes were inactivated by adding 4 ml of PBS. Cell suspensions were centrifuged for 5 min at 1500 rpm at 4 °C. The spinal cords were mechanically homogenized, and the debris was removed by passing through a 70-μm cell strainer. Finally, the cells were centrifuged for 5 min at 1500 rpm at 4 °C. To remove any potential contamination of myelinated cells, a myelin removal protocol was used. The cell pellet was resuspended in 1200 μl of Myelin Removal Bead Mix (120 μl Myelin Removal Beads and 1080 μl 0.5% BSA PBS). The cells were incubated for 15 min on ice. MACS LS columns were primed with 0.5% BSA/PBS and the cell suspension in 0.5% BSA/PBS was added to the MACS LS column. For viability, the Invitrogen Fixable Viability Dye eFluor 780 (Thermo Scientific, 65–0865-18) was added in the antibody mix. The flow-through was collected and the columns were washed with 0.5% BSA/PBS. The flow-through containing non-myelinated spinal cells was spun down at 500 g for 15 min at 4 °C.

Cell number and viability were determined using an automated counting method (Cellometer Auto 2000 Cell Viability Counter Profiler, Nexcelom Bioscience). Cells were suspended in flow cytometry buffer (Thermo Scientific,50–112–9748) at a concentration of 5 × 10^6 cells and blocked with 1 µg Fc Block (BD Biosciences, 550270) for 15 min. Cells were then stained with multi-color fluorophore-conjugated antibodies on ice for 30 min and washed twice with 1% BSA/PBS/0.02% sodium azide. The fluorescently conjugated antibodies used to detect microglia were CD11b/c FITC and CD45APC (Thermo Scientific, 17–0461-82) and CD86 PE antibody (BD Biosciences, 551396) was used as a marker of activation. All labeled cell suspensions were fixed in 1% paraformaldehyde and stored at 4 °C in the dark until analyzed by flow cytometry. Cells were analyzed using a BD LSR II (BD Bioscience) and FlowJo software (FlowJo, LLC, Ashland, CA).

### Statistical analysis

The differences between conditions (CCI sham controls, CCI + tsFUS and CCI + tsFUS sham) within each group were assessed by ANOVA or a non-parametric test for non-normal distribution (ranksum, MATLAB R2021a, The MathWorks, Inc., USA). The significance level was set at 0.05. All data analyses were performed using MATLAB (R2021a, The Math Works, Inc., USA).

## Results and discussion

### Trans-spinal FUS after CCI reduces mechanical sensitivity in a sustained manner

**Fig. 2:**
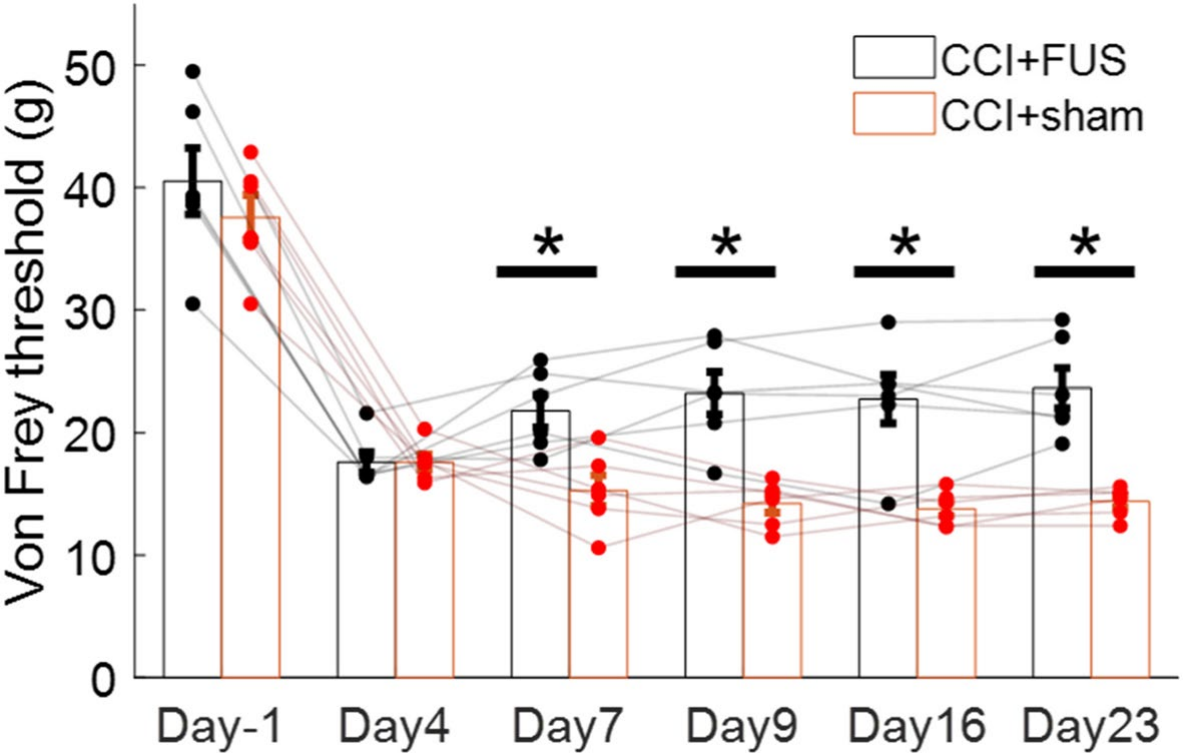
Treatment with trans-spinal FUS sustainably reduces sensitivity to pain after chronic constriction injury (CCI). Pain sensitivity was assessed using the Von Frey threshold (VFT). VFT was measured before CCI (day −1), after CCI (day 4), after the end of 3-day tsFUS or sham treatment (day 7), and on several follow-up timepoints (days 9, 16, 23). After CCI (day 4), VFT decreases in both the sham and tsFUS-treated animals. At days 7, 9, 16, and 23 after CCI, tsFUS-treated animals show reduced VFT compared to sham-treated animals. Each dot represents an individual animal. Error bars represent mean ± S.E. t-test (*n* = 12; *p < 0.05)

The von Frey test is used in rodents to assess mechanical sensitivity and evaluate the effectiveness of analgesic treatments (Deuis et al. 2017). We measured withdrawal threshold to mechanical stimuli in the left paw, before and at different times after CCI of the sciatic nerve in animals that received tsFUS or sham tsFUS stimulation. As expected, withdrawal threshold decreased after CCI compared to baseline. In sham-treated animals, withdrawal threshold remained decreased for at least 23 days. In animals treated with tsFUS the withdrawal threshold began to recover on the day after treatment ended (day 4 after CCI) and remained decreased for the reminder of the observation period, up to 23 days later (Fig. 2). Although we did not perform a formal histological safety evaluation in this study, our pilot test in a healthy rat showed no sign of tissue damage following the same tsFUS protocol (sup Fig. 1). Additionally, in this study, we monitored the animals daily post-therapy for any signs of additional posture changes.

### Trans-spinal FUS after CCI reduces the number of spinal microglia ipsilateral to injury

**Fig. 3:**
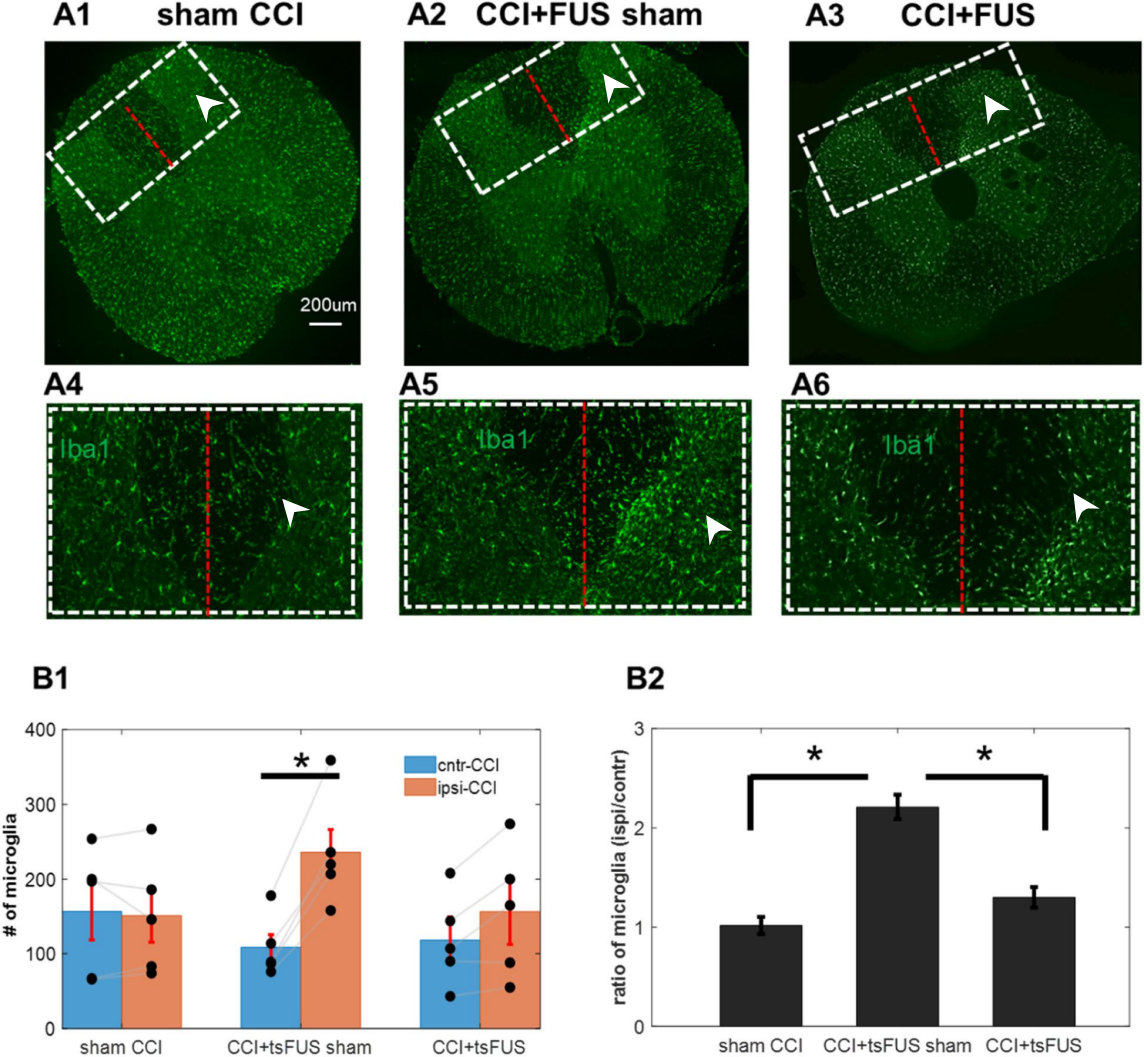
Treatment with tsFUS in CCI animals reduces numbers of spinal microglia ipsilateral to injury. **A** Example IHC images showing microglia on spinal sections at L5 level labeled with Iba1. A1: Sham CCI animal, A2: CCI animal that received sham stimulation (CCI + sham FUS). A3: CCI animal that received tsFUS (CCI + tsFUS). The white box in each panel shows the region of interest (ROI) used to quantify microglia, separately for each side of the spinal cord; red line represents midline. A4-A6: Rotated and magnified images corresponding to ROIs from respective panels. Arrow indicates ipsilateral side to the CCI in each panel. **B** Quantification of the effect of CCI and tsFUS on number of spinal microglia. B1, number of microglia within the ROI, reported separately for contralateral and ipsilateral sides to the CCI, in sham CCI, CCI + FUS sham, and CCI + tsFUS animals. Each symbol represents an individual animal (paired t-test; *p* < 0.05 is deemed significant). B2: Ratios of the number of microglia on the ipsilateral to contralateral sides (ANOVA with group as independent variable, followed by pair-wise comparisons between groups; *p* < 0.05 corrected for multiple comparisons is deemed significant)

Microglia play an important role in the development of NP (Costigan et al. 2009). We identified microglia from the L5 segment by IHC staining with the widely used marker IBA1 (Fig. 3, A). Since cells in the dorsal horns are affected in painful syndromes (Todd 2010), we determined the number of microglia within a dorsal region of interest (ROI) on both sides of the spinal segment. As expected, we found no significant difference in the number of microglia between the two sides of the spinal cord in sham CCI animals (Fig. 3, A1, A4, B1). After CCI with sham FUS, elevated numbers of microglia were observed in the ipsilateral compared to the contralateral CCI side, which suggests inflammation in the ipsilateral side activated more microglia in in the contralateral side than the ipsilateral side of spinal cord to the injury. (Fig. 3, A2, A5, B1, B2). In contrast, tsFUS treatment reduced the increase in microglia in the ipsilateral side (Fig. 3, B1 and B2), and reduced the ipsi/contra ratio of microglia (Fig. 3 B2). This indicates that FUS might reduce the inflammation of the injury.

### Trans-spinal FUS after CCI suppresses activation of microglia

**Fig. 4:**
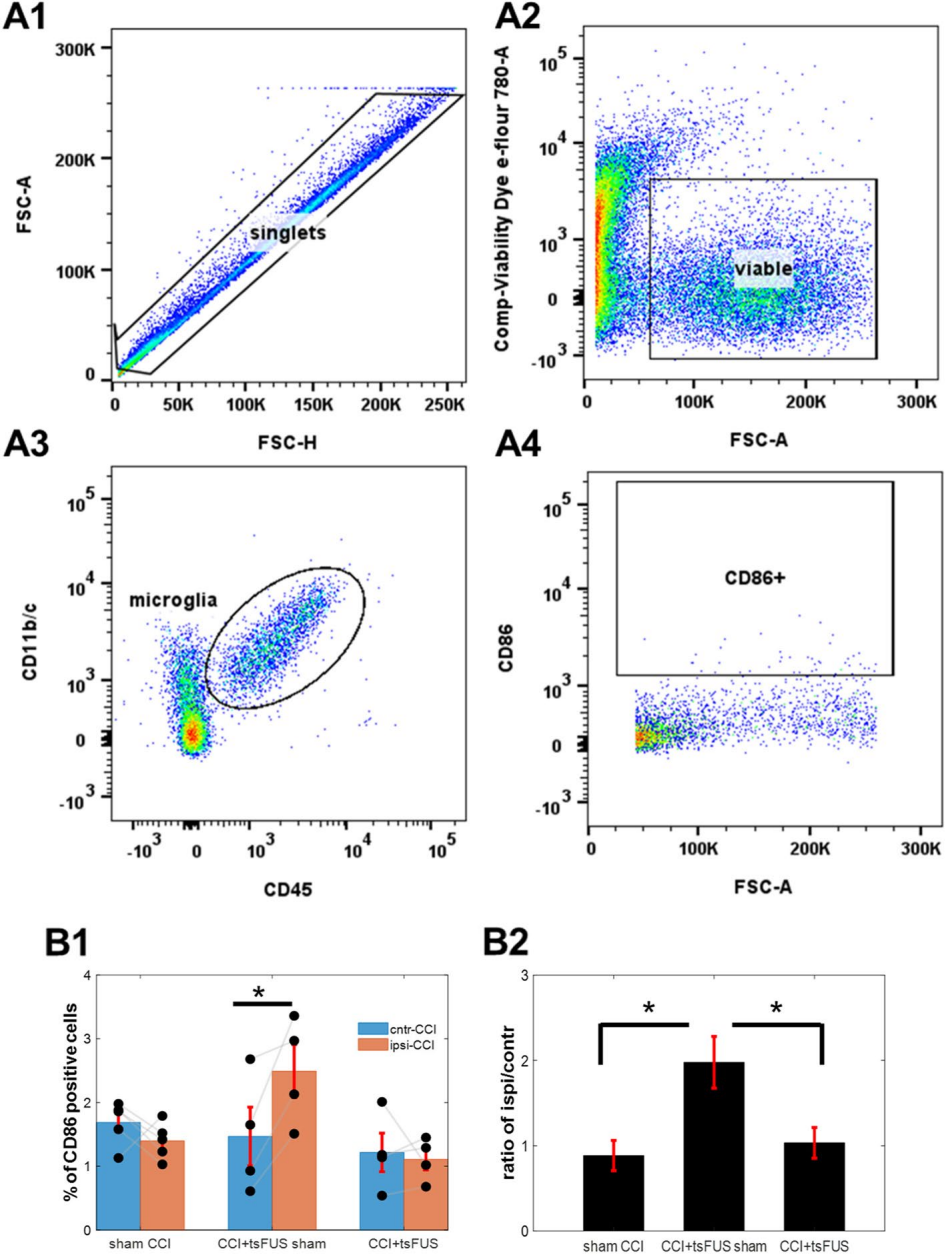
Treatment with tsFUS in CCI animals reduces activated spinal microglia, ipsilateral to injury. **A** Flow cytometry gating strategy to detect activated spinal microglia. A1: Cells were first gated on singlets. A2: Only viable cells from singlets were analyzed. A3: Microglia were identified using the cell surface markers CD45 and CD11b/c + . A4: The activated of microglia were identified by CD86 expression (a biomarker of M1 phenotype). **B** Effect of CCI and tsFUS on number of activated spinal microglia. B1, Number of activated microglia from spinal segments L4 and L6, shown separately for the contralateral and ipsilateral sides to the CCI, in each testing group. Each dot represents individual rat. (t-test; *p* < 0.05). B2: Ratios of ipsilateral to contralateral counts of activated microglia. (ANOVA; *p* < 0.05)

Spinal microglia exist in different phenotypes (Wendimu and Hooks 2022), and a pro-inflammatory phenotype is strongly associated with development of NP. From cell homogenates isolated from ipsilateral and contralateral sides of the spinal cord at the L4-L6 segments, we quantified activation of spinal microglia using flow cytometry (Fig. 4, A1-A4). In sham CCI animals, we found no significant difference in the number of microglia in the ipsilateral and contralateral sides of the spinal cord (Fig. 4 B1). In animals with CCI treated with sham tsFUS, activated microglia were increased in the ipsilateral side compared to the contralateral side (Fig. 4 B1). Notably, in animals with CCI treated with tsFUS, the number of activated microglia on the ipsilateral side were reduced. The ipsi/contra ratio of activated microglia number was significantly increased in CCI animals treated with sham tsFUS compared to sham CCI animals, while treatment with tsFUS restored the ratio to that observed in sham CCI animals (Fig. 4 B2). In this study, we used microglial biomarkers (Iba1 and CD86) to identify activated microglia. We found suppression of activated microglia by tsFUS only in the insonified side, suggesting a localized effect. This is consistent with our previous findings of a localized, segment-specific effect of tsFUS on spinal circuit excitability (Song et al. 2024).

Tran-spinal FUS modulates spinal circuit function with potentially improved resolution and penetration depth, attributes that are potentially promising therapeutically in NP (Song et al. 2024). In this study, we find that CCI causes a significant reduction in mechanical withdrawal threshold and treatment with tsFUS increased this threshold compared to sham tsFUS-treated animals (Fig. 2). The behavioral effect of tsFUS was sustained for weeks after treatment (Fig. 2). After CCI, microglia were increased in number and activated on the ipsilateral side. tsFUS significantly reduced both the number (by IHC) and their activation of microglia showing CD86 positive (by flow cytometry) (Figs. 3 and 4).

NP is a complex condition with a range of causes and underlying mechanisms(Campbell and Meyer 2006), among which spinal microglia play a central role. Microglia after CCI are activated into pro-inflammatory (M1) and anti-inflammatory (M2) phenotypes, releasing mediators that exert opposing effects. Microglia activation was found to begin two days after CCI to the sciatic nerve, peaking between days seven and nine (Mika et al. 2009). In our study, spinal cord tissue was collected 7 days after CCI and as expected, microglia were elevated in the ipsilateral (to the nerve injury) spinal cord (Fig. 3). These microglia were activated, as indicated by CD86 (mostly used as M1 phenotype marker) levels detected by flow cytometry (Fig. 4). Others have shown that activated M1 microglia release pro-inflammatory substances such as interleukin (IL)−6, IL-1β, and tumor necrosis factor-α (TNF-α) and their pharmacological inhibition substantially reduces nerve injury-induced mechanical allodynia (Schafers et al. 2003). Our finding that tsFUS reduces CD86 positive microglia, while at the same time reducing mechanical sensitivity, is consistent with a possible direct anti-inflammatory effect of FUS on microglia. FUS has been used to modulate microglial structure and function (Grewal et al. 2023), and low-intensity FUS was found to reduce microglia-mediated neuroinflammation following the implantation of microelectrodes (Li et al. 2024).

**Fig. 5:**
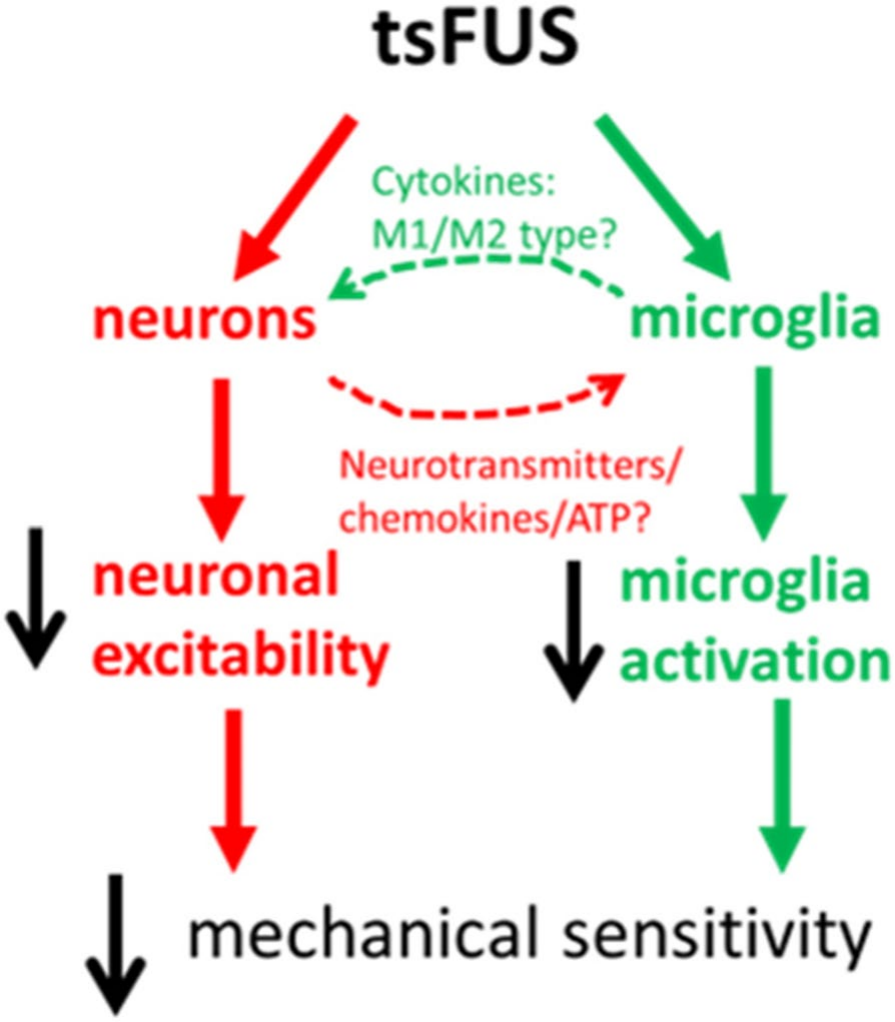
The schematic diagram of the potential mechanism of tsFUS on pain control. tsFUS suppresses pain could be directly on neurons to decrease the excitability (red) or indirectly through reducing the activation of microglia (green)

Another major pathogenic mechanism for the development of neuropathic pain is abnormal neuroplasticity resulting in pathological increase in excitability of primary sensory neurons and nociceptive neurons in the spinal cord and brain (Campbell and Meyer 2006). Suppressing over-excitability of these neurons has been used to relieve pain (Hiraga et al. 2022) and FUS targeting dorsal root ganglia has been found to increase threshold for noxious mechanical stimuli (Liss et al. 2021). FUS modulates neural activity through different mechanisms, including mechanical and thermal energy (Guo et al. 2022). It was demonstrated that increased neural activity after CCI was required for the development of NP (Xie et al. 2005), and CCI causes activation of the microglia of the spinal cord (Nishihara et al. 2020). Neuronal activity via the stimulation of microglia could feed back to inhibit neuronal networks (Badimon et al. 2020). We recently found that in healthy rats, tsFUS directly suppresses monosynaptic H-reflex and polysynaptic flexor reflexes, indicating effects on excitability and synaptic transmission(Song et al. 2024). In the present study, the decrease in pain sensitivity from tsFUS may be due to direct effect of FUS on neurons, resulting in reduced abnormal excitability of spinal neurons or altered synaptic transmission along with the suppression of the inflammation (Fig. 5). The effect on excitability may also include indirect mechanisms: microglia have complex interactions with neurons, and are powerful neuromodulators that regulate synaptic transmission (Brennan et al. 2024), leading to increased neuronal excitability, central sensitization and decreasing inhibition from interneurons. Finally, direct or microglia-mediated effects of FUS on pain-associated synaptic plasticity cannot be ruled out (Campbell and Meyer 2006).

### Study limitations

Out study has several limitations. First, we examined tsFUS only in the early phase of NP. Since NP initiation, rather than maintenance, is primarily linked to immune abnormalities (Xu et al. 2006), tsFUS may engage different mechanisms depending on treatment timing (Campbell and Meyer 2006).Second, we tested tsFUS only in adult male rats. It is known that sex and age have major impact on pain perception (Bodnar et al. 1988; Mogil and Bailey 2010). The use of male animals, which generally have reduced variability in pain study outcomes [provide refs], may be justified in this study by the fact that this is an initial investigation of a relatively new neuromodulation approach, and serves as proof of concept for additional mechanistic and preclinical studies. Third, we only tested one set of tsFUS parameters (PRF, duty cycle, mechanical index, duration and localization). Much of the parameters choices were made based on our prior work, which documented spinal segment-specific effects of tsFUS on spinal circuit excitability without any histological evidence for tissue damage, at least after a single tsFUS session (Song et al. 2024). Fourth, we conducted relatively limited characterization of the microglial response to CCI and to tsFUS, as well as their impact on astrocytes and other immune cells in the spinal cord and relation to changes in neuronal excitability and plasticity (Hiraga et al. 2022; Brennan et al. 2024). Future studies will document the impact of sex, age, stimulation parameters on this potentially clinically interesting effect of tsFUS and will investigate its long-term efficacy, safety and neuroimmune and in-depth mechanistic basis. Fifth, our study lacks additional control groups, such as animals that did not undergo CCI but received tsFUS treatment, or animals that underwent CCI at nerves affecting different spinal segments than those that were insonified. Future control experiments will help investigate the effects of tsFUS on normal spinal physiology, beyond its use in CCI animals, which is the focus of this *Brief Report*. Lastly, differences between species must be considered when translating rodent findings to human pain treatments. A major difference is the increased thickness of bone structures in the human spinal column, which may limit the amount of mechanical energy delivered to the spinal cord to sub-therapeutic levels, something that may be mitigated with longer, more intense insonification.

## Conclusion

Non-invasive neuromodulation methods hold promise for treating disorders of the spinal cord, including NP and spinal cord injury (Hong et al. 2022). In contrast to previous FUS neuromodulation of DRGs (Hellman et al. 2021), we directly target spinal microglia and spinal neurons to treat NP at the developing phase. While the exact mechanism is unclear, our study shows that early tsFUS treatment of CCI modulates microglia activation and reduces mechanical hypersensitivity. These results support further investigation into the therapeutic potential for modulating circuits involved in pain-related disorders or spinal cord injury.

## Supplementary Information

Supplementary Material 1

## Data Availability

The datasets used are available from the corresponding author on reasonable request.

## Abbreviations

NP: Neuropathic pain
CCI: Chronic constriction injury
tsFUS: Trans-spinal focused ultrasound stimulation
PRF: Pulse repetition frequency
