# Supplementary material for: Low intensity trans-spinal focused ultrasound reduces mechanical sensitivity and suppresses spinal microglia activation in rats with chronic constriction injury
Source: Bioelectron Med. 2025 Mar 31;11:8. doi: 10.1186/s42234-025-00170-z (PMC11956222; doi:10.1186/s42234-025-00170-z)
Supplement: Supplementary file 1 — Supplementary Material 1 [file 42234_2025_170_MOESM1_ESM.pdf]

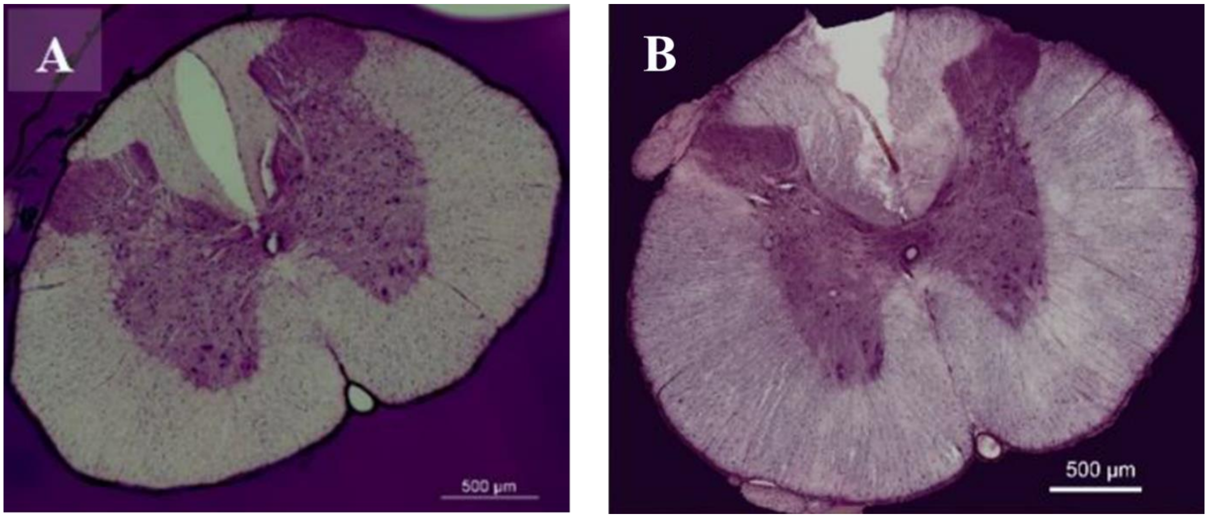

Supp figure 1. Hematoxylin and Eosin staining of the L5 spinal segment in a rat subjected to tsFUS (A) and a rat receiving sham tsFUS (B).
